# Cassiae semen extract ameliorates hyperlipidemia in rats by modulating lipid metabolism and FcγR-mediated immune regulation

**DOI:** 10.3389/fphar.2025.1546119

**Published:** 2025-04-28

**Authors:** Mingyue Lv, Yannan Zheng, Man Yuan, Errui Zhang, Min Zheng, Guangyao Liu, Min Zheng, Weiliang Gu, Hongxi Xu

**Affiliations:** ^1^ School of Pharmacy, Shanghai University of Traditional Chinese Medicine, Shanghai, China; ^2^ Engineering Research Center of Shanghai Colleges for TCM New Drug Discovery, Shanghai, China; ^3^ The Institute of Chinese Materia Medica, Shanghai University of Traditional Chinese Medicine, Shanghai, China; ^4^ Shuguang Hospital Affiliated to Shanghai University of Traditional Chinese Medicine, Shanghai, China

**Keywords:** cassiae semen extract, hyperlipidemia, gut microbiota, lipid metabolism, fc gamma R, high-fat diet, inflammation

## Abstract

**Introduction:**

Cassiae Semen Extract (CSE) shows promise in treating hyperlipidemia, although its underlying mechanisms are not yet fully understood. This study aimed to investigate the effects of CSE on hyperlipidemia in rats and explore the potential mechanisms involved.

**Methods:**

Hyperlipidemic rats were induced by a high-fat diet (HFD) and treated with CSE. Serum, liver, and fecal samples were analyzed through biochemical assays, histopathological examination, 16S rRNA sequencing, KEGG pathway analysis, and Western blot.

**Results:**

CSE treatment effectively alleviated biochemical imbalances and tissue damage induced by the HFD. 16S rRNA sequencing revealed that CSE improved gut microbiota dysbiosis and increased microbiota abundance. Pathological analysis showed that CSE reduced hepatic lipid accumulation, mitigating liver damage. KEGG pathway analysis suggested that the beneficial effects of CSE on hyperlipidemia may involve Fc gamma receptor (FcγR)-mediated phagocytosis, with immune activation influencing lipid homeostasis and liver inflammation. Western blot analysis further indicated that CSE may regulate lipid metabolism via Sterol Regulatory Element-Binding Protein-1c (SREBP-1c) and Peroxisome Proliferator-Activated Receptor Alpha (PPARα), while reducing hepatic inflammation through the MAPK signaling pathway.

**Discussion:**

CSE may ameliorate hyperlipidemia in rats by modulating gut microbiota disorders, lipid metabolism, and FcγR-mediated immune regulation, providing a potential therapeutic approach for diseases associated with metabolic dysfunction and inflammation. However, further in-depth studies are required to fully elucidate these mechanisms.

## 1 Introduction

Hyperlipidemia, also known as dyslipidemia, is one of the significant factors contributing to atherosclerosis. Changes in dietary habits and lifestyle have led to an annual increase in its prevalence, largely due to excessive intake of high-fat foods (Chang et al., 2021). This condition is typically characterized by elevated levels of serum total cholesterol (TC), triglycerides (TG), low-density lipoprotein cholesterol (LDL-C), and decreased levels of high-density lipoprotein cholesterol (HDL-C) (Nelson, 2013; Liu et al., 2024). Managing lipid levels is essential in addressing this health challenge. While regular exercise and dietary adjustments are fundamental, medications are often necessary. However, effective medications with minimal side effects remain scarce. Therefore, the development of efficacious lipid-lowering drugs is crucial.

Currently, statins serve as the primary agents for managing hyperlipidemia. Apart from common side effects such as intolerance and treatment resistance observed in clinical practice (Tsushima and Hatipoglu, 2023), recent studies have indicated that certain statins may elevate the risk of muscle-related issues due to drug interactions (Newman, 2022; Lamprecht et al., 2022). Compared to conventional lipid-lowering drugs, the principal advantages of CSE predominantly lie in its minimal toxic profile. Furthermore, CSE holds dual utility as both a medicinal remedy and dietary supplement, underscoring its safety and efficacy as a treatment option (Ng et al., 2023).

Nowadays, the integration of gut microbiota analysis alongside non-targeted metabolomics analysis of drugs for treating metabolic disorders has become increasingly widespread (Li and Wang, 2023). The stability of the body’s physiological state can be assessed through gut microbiota analysis. In addition to the most abundant phyla such as Bacteroidetes and Firmicutes (Tawulie et al., 2023; Jandhyala et al., 2015), alterations in dietary patterns and the onset of diseases can disrupt the composition of the gut microbiota, resulting in dysbiosis (Vourakis et al., 2021). Reports suggest that traditional Chinese medicine can achieve the purpose of treating certain metabolic diseases by regulating the composition and levels of intestinal microbes (Wang et al., 2023). For instance, Cheng et al. (2023) studied how retrograded rice enhances the abundance of propionic acid-producing bacteria, which inhibit cholesterol synthesis and thus lower cholesterol levels. Similarly, Li et al. (2022) demonstrated that polysaccharides can increase beneficial bacteria, decrease harmful bacteria, and ameliorate hyperlipidemia. Various metabolites of gut microbiota are intricately linked to blood lipid levels (Jia et al., 2021), and previous studies have underscored the therapeutic potential of CS on hyperlipidemia (Mao et al., 2022; Yuen et al., 2021). Nevertheless, comprehensive investigations are still needed.

In this study, a hyperlipidemic rat model was established to assess the lipid-lowering effects of CSE. 16S rRNA sequencing was used to evaluate the impact of CSE on gut microbiota composition and abundance. Additionally, nontargeted metabolomics was applied to explore the molecular mechanisms underlying CSE’s effect on lipid metabolism.

## 2 Materials and methods

### 2.1 Experimental materials and reagents

Cassiae Semen Extract (CSE) was purchased from Shaanxi Jiahe Phytochem Co., Ltd. (CJMZ-A-A036586). Alanine aminotransferase (ALT/GPT) test kit, aspartate aminotransferase (AST/GOT) test kit, high-density lipoprotein cholesterol (HDL-C) test kit, low-density lipoprotein cholesterol (LDL-C) test kit, total cholesterol (TC) test kit, and triglyceride (TG) test kit were purchased from Nanjing Jiancheng Bioengineering Institute. Malondialdehyde (MDA) content detection reagent kit was purchased from Sangon Biotechnology (Shanghai) Co., Ltd. Anti-PPARα antibody, anti-SREBP-1c antibody were purchased from Santa Cruz Biotechnology Co., Ltd.; Anti-ERK antibody, anti-p-ERK antibody were purchased from Cell Signaling Technology Co., Ltd.

### 2.2 Preparation and quality evaluation of CSE

#### 2.2.1 Preparation of CSE

Cassiae Semen (*Cassia tora* L., Fabaceae) was authenticated by Dr. Xupeipei (Shaanxi Jiahe Phytochem Co., Ltd.) according to the *Chinese Pharmacopoeia* (Part 1). The decoction piece was extracted three times with 60% ethanol, each extraction lasting 1.5 h. The consequent extracting solution was combined, concentrated and then spray-dried to obtain a powder (CSE).

The quality of CSE were evaluated with thin-layer chromatography (TLC), high-performance liquid chromatography (HPLC), and high-performance thin-layer chromatography (HPTLC) methods by validating its identity, purity, and consistency through comparison of the chromatographic profiles of the samples, reference standards, and controls. The integrated analytical workflow ensures comprehensive quality control of CSE.

#### 2.2.2 TLC identification

To prepare the sample solution, 3 g of CSE was macerated in 10 mL of methanol for 1 h, filtered, and then evaporated to dryness. The obtained residue was dissolved with 10 mL of water, acidified with 1 mL of hydrochloric acid, and heated in a water bath for 30 min. After cooling, the solution was extracted twice with 20 mL of diethyl ether. The ether layers were combined, dried, and reconstituted in 1 mL of chloroform, then the sample solution was obtained. The negative control solution was prepared by following the same procedure but omitting CSE to ensure specificity. A mixed reference solution of aurantio-obtusin and chrysophanol (1 mg/mL respectively) was prepared by dissolving the reference substances with a mixture (2:1, *v/v*) of absolute ethanol and ethyl acetate.

For the TLC identification, 2 μL of the sample solution, reference solution, and negative control solution were spotted onto a pre-activated silica gel H plate. The plate was developed using a petroleum ether (30°C–60°C)-acetone (2:1, *v/v*) solvent mixture, dried, and visualized. Then the plate was revisualized after it was exposed to ammonia vapor (after fumigation).

#### 2.2.3 HPLC analysis

For the sample solution preparation, 1.0 g of CSE was ultrasonicated in 10 mL of 70% ethanol for 60 min and then filtered using a 0.45 μm membrane. For the reference standard solution, aurantio-obtusin and obtusifolin (0.5 g each) were dissolved in 5 mL of 70% ethanol, ultrasonicated for 60 min, and filtered. The HPLC analysis was developed using an Agilent 1200 HPLC system with a DAD detector set to 254 nm. The column was a CAPCELL PAK ADME (4.6 × 150 mm, 3 μm). The mobile phase consisted of two solutions, A (0.1% formic acid in water) and B (0.1% formic acid in acetonitrile). The gradient program was as follows: at 0 min, A 95% and B 5%; at 58 min, A 30% and B 70%; and at 60 min, A 95% and B 5%. The flow rate was set to 1.0 mL/min, with an injection volume of 10 μL and the column temperature maintained at 30°C.

#### 2.2.4 HPTLC identification

For the sample solution preparation, 0.5 g of CSE was ultrasonicated in 5 mL of 70% ethanol for 30 min and filtered. For the raw material reference preparation, 1.0 g of Cassiae Semen was ultrasonicated in 10 mL of 70% ethanol for 60 min and then filtered. HPTLC was conducted using a stationary phase of silica gel 60. The loading volume was 4 μL. The developing phase consisted of ethyl acetate, formic acid, acetic acid, and water in a ratio of 10:1:1:2 (*v/v/v/v*). The development was performed with 6 cm migration after 15 min of chamber saturation. After the developing, the plate was dried in the air, color-developed with 10% sulfuric acid-ethanol solution, heated at 105°C until the spots were clear, and then examined under a ultraviolet light (366 nm).

### 2.3 Animal grouping

In this study, a total of 40 male Wistar rats were purchased from Shanghai SLAC Laboratory Animal Co., Ltd. All animal experimental procedures were performed following the guidelines for the care and use of laboratory animals at Shanghai University of Traditional Chinese Medicine, with an ethical approval number of PZSHUTCM2304170005. The rats were housed under a 12-h light/dark cycle at a temperature of 22°C ± 2°C and a relative humidity of 50%∼70%. After a 7-day adaptation period, 8 rats were randomly selected and fed a normal diet as the normal group (abbreviated as N group), while the remaining 32 rats were fed a high-fat diet (HFD), formulated with additional 20.0% sucrose, 15% lard, 1.2% cholesterol, 0.2% sodium cholate, and appropriate amounts of casein, calcium hydrogen phosphate, and stone powder, to induce a high-fat model. After 3 weeks of gavage feeding, blood samples were collected from the orbital vein to obtain serum. The levels of total cholesterol (TC), triglycerides (TG), high-density lipoprotein cholesterol (HDL-C), and low-density lipoprotein cholesterol (LDL-C) in the serum were measured using an automatic biochemical analyzer to confirm the establishment of the high-fat model, evidenced by elevated TC, TG, and LDL-C levels, and reduced HDL-C levels (Hu et al., 2021). The rats in the HFD group were then randomly divided into four groups: the model group (M group) and three CSE-treated groups based on dose, which are low-dose CSE group (CSL, 50 mg/kg/day, Human Equivalent Dose (HED) = 2 g/day, 100 mg CSE dissolved in 20 mL sterile water), medium-dose CSE group (CSM, 100 mg/kg/day, HED = 4 g/day, 200 mg CSE dissolved in 20 mL sterile water), and high-dose CSE group (CSH, 200 mg/kg/day, HED = 8 g/day, 400 mg CSE dissolved in 20 mL sterile water). Rats in the N group and M group received gavage of sterile water, while the other groups received corresponding doses of CSE. The intervention lasted for 60 days. Following the intervention, animals were anesthetized with an intraperitoneal injection of 50 mg/kg Zoletil, and blood and tissue samples were collected.

### 2.4 Measurement of body weight and liver index

Body weight of the rats was recorded every 3 days during the administration period. After 60 days of treatment, the rats were euthanized, and their livers were carefully dissected. The livers were rinsed in PBS, blotted dry with absorbent paper to remove excess moisture, and weighed immediately. The right lobe was clipped and fixed in 4% paraformaldehyde, while the remaining liver tissue was rapidly preserved in liquid nitrogen for further analysis. The liver index was calculated using the following formula:

$$\text{Liver index }\left(\%\right)=\text{Liver weight }\left(g\right)\;/\text{ Rat weight }\left(g\right)\times 100\%$$

### 2.5 Determination of biochemical indicators

Before euthanasia, rats were fasted for 12 h with unrestricted access to water. Blood samples were collected from the abdominal aorta and left to stand for 1 h. After centrifugation at 3,000 rpm for 15 min at 4°C, serum was separated to measure levels of TC, TG, HDL-C, LDL-C, AST, ALT, and MDA. Liver homogenates were prepared to determine levels of TC and TG.

### 2.6 Hematoxylin and eosin staining (H&E staining)

Rat liver tissue was fixed in 4% paraformaldehyde for 24 h, followed by paraffin embedding. The sections were stained with hematoxylin and eosin, and the pathological condition of the liver tissue was observed under an optical microscope.

### 2.7 Oil Red O staining

Liver tissue samples fixed in 4% paraformaldehyde were initially washed with PBS, embedded for frozen sections, and stained with Oil Red O working solution for 20 min. After rinsing with PBS for 30 s, the sections were counterstained with hematoxylin to visualize cell nuclei, and then mounted with glycerol for microscopic observation.

### 2.8 Analysis of rat gut microbiota

Rat fecal samples were collected and stored at −80°C for later use. Genomic DNA was extracted from the feces of each group by FastPure Stool DNA Isolation Kit (MJYH, shanghai, China), and then examined using 1% agarose gel electrophoresis. Specific amplification of the V3-V4 region of the 16S gene was performed using a pair of universal primers (338 F and 806 R) (Zhou et al., 2019). Sequencing analysis was conducted on the Illumina Nextseq 2000 platform provided by Shanghai Majorbio Bio-pharma Technology Co., Ltd. (Shanghai, China). Paired-end reads (PE reads) obtained from Illumina sequencing were assembled based on overlapping relationships, followed by quality control and sequence filtering. Operational Taxonomic Unit (OTU) clustering and taxonomic classification were performed based on 97% similarity (http://www.drive5.com/uparse/). Alpha diversity analysis assessed microbial community richness and diversity, while beta diversity analysis explored community structure similarities or differences. Species composition analysis investigated changes in microbial communities at the phylum, family, and genus levels, alongside hierarchical differential species analysis.

### 2.9 Metabolomic analysis

The cecal contents of each group were collected and stored at −80°C. LC-MS was employed for untargeted metabolomic analysis of the samples. Samples were extracted with methanol and centrifuged at 13,000 rpm for 15 min at 4°C. The supernatant metabolites were analyzed using LC-MS at Majorbio. Data underwent subsequent processing and bioinformatics analysis. Principal component analysis (PCA) was conducted using ropls (R packages, Version 1.6.2) to assess overall metabolic differences. Differential metabolites were classified using metabolites clustering analysis (KEGG kegg_v20221012) and KEGG enrichment pathways were analyzed to explore potential mechanisms underlying the lipid-lowering effects of CSE.

### 2.10 Western blot

An appropriate amount of RIPA lysate was added to each animal tissue, which was milled with a grinder and fully lysed. The protein concentration of each sample was determined using BCA kit. Protein separation was performed using the Omni-Easy™ One-Step PAGE Gel Rapid Preparation Kit, and the separated proteins were transferred to a PVDF membrane, which was closed with 5% skimmed milk powder at room temperature for 1.5 h. Anti-PPARα antibody (Santa cruz, sc-398394), anti-SREBP-1c antibody (Santa cruz, sc-13551), anti-ERK antibody (Cell Signaling Technology, 4,695), anti-p-ERK antibody (Cell Signaling Technology, 4,370) were diluted 1:1,000, and incubated at 4°C overnight. Secondary antibodies were diluted 1:5,000 and incubated at room temperature for 1.5 h before visualization using an imaging system, followed by grey scale analysis using IMAGE J software.

### 2.11 Statistical analysis

Experimental data were expressed as mean ± standard deviation (mean ± SD). All data were analyzed using GraphPad Prism 8 statistical software. One-way analysis of variance (ANOVA) was used for variance analysis among multiple groups, with statistical significance set at p < 0.05.

## 3 Results

### 3.1 Quality evaluation of CSE

**FIGURE 1:**
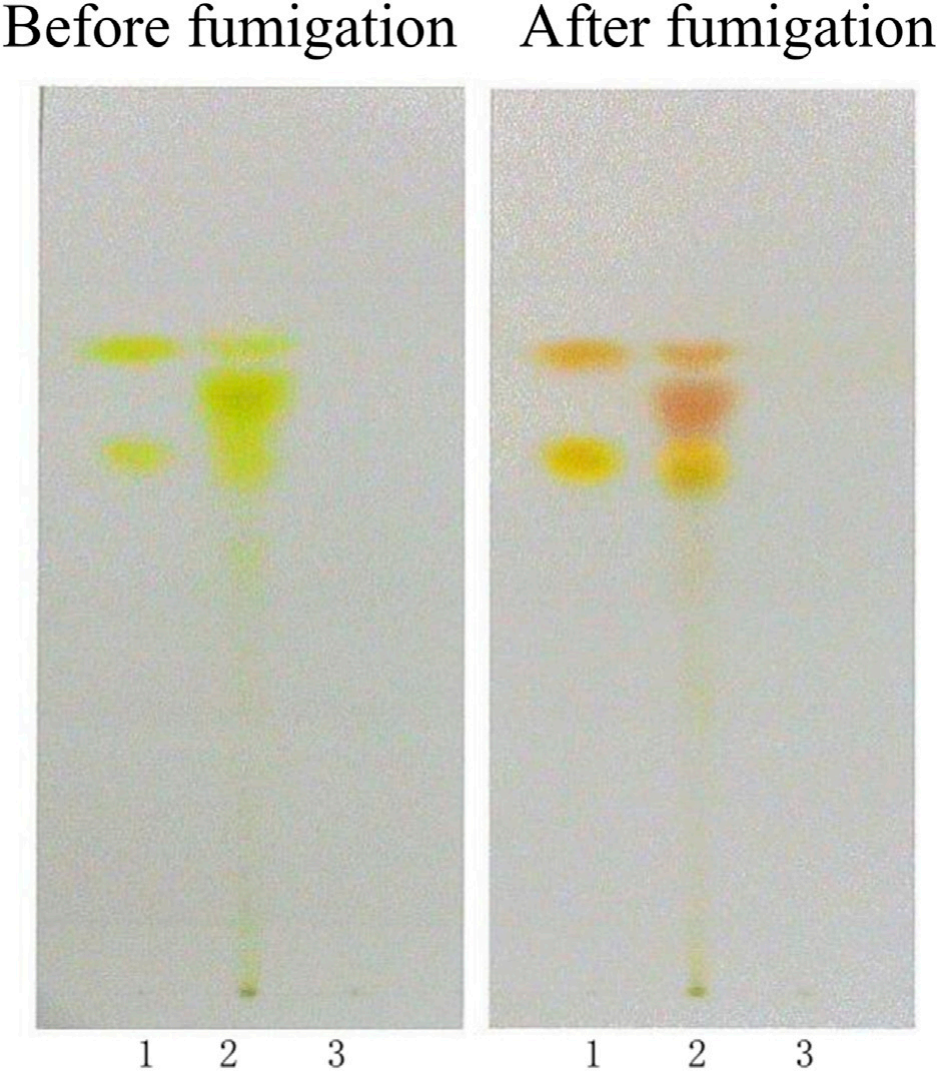
TLC identification of CSE. (1. Aurantio-obtusin, chrysophanol; 2. Sample; 3. Negative control sample).

In the chromatogram of the sample solution, the spots appeared at the same positions as those in the reference standard chromatogram, exhibiting identical coloration. After exposure to ammonia vapor, these spots turned bright yellow (aurantio-obtusin) and pink (chrysophanol). The negative control chromatogram showed no detectable spots at the corresponding positions of the reference standards. Following ammonia vapor treatment, no yellow (aurantio-obtusin) or pink (chrysophanol) spots were observed. No interference was detected in the negative control sample during the identification process. The TLC results were shown in Figure 1.

**FIGURE 2:**
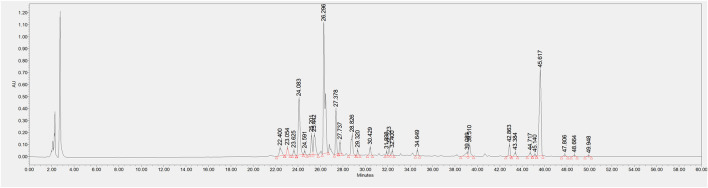
HPLC chromatogram of CSE.

The HPLC chromatograms of the samples displayed characteristic peaks in CSE. It showed that the retention time for aurantio-obtusin was approximately 43.384 min, while obtusifolin had a retention time of around 47.806 min. The retention times and peak shapes matched those of the reference material. See Figure 2.

**FIGURE 3:**
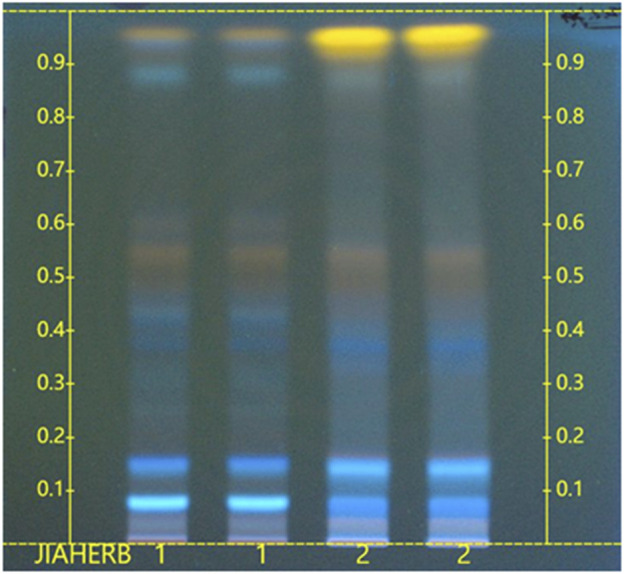
HPTLC chromatogram of CSE. (1. Raw material standard (NIFDC, 121544-202002); 2. CSE sample.)

In Figure 3, lane 2 shows the CSE used in this experiment, while lane 1 represents the raw material standard for comparison. The chromatographic profile of the test sample was consistent with that of the reference standard.

### 3.2 Effects of CSE on rat physiological functions

**FIGURE 4:**
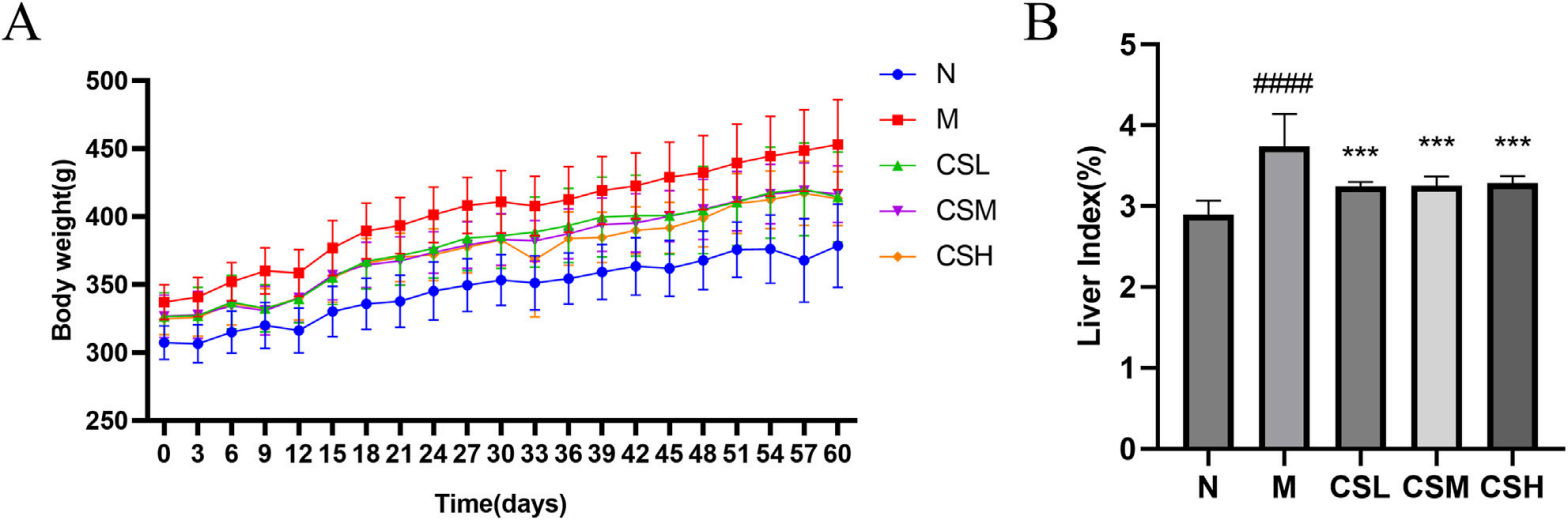
Effects of CSE on body weight and liver index in hyperlipidemic rats. **(A)** Body weight, **(B)** Liver index. Values were presented as mean ± SD (n = 8). vs. Group N: ####p < 0.0001; vs. Group M: ***p < 0.001.

Figure 4A illustrates the changes in body weight of hyperlipidemic rats following intervention with CSE. Rats in the HFD group exhibited a significant increase in body weight compared to the N group (p < 0.01). However, this abnormal weight gain was mitigated with CSE intervention. Additionally, the liver index of rats in the M group showed a significant increase compared to the N group (p < 0.0001). Treatment with CSE effectively attenuated this abnormal liver growth (p < 0.001) (Figure 4B).

### 3.3 Effects of CSE on serum biochemical parameters in hyperlipidemic rats

**FIGURE 5:**
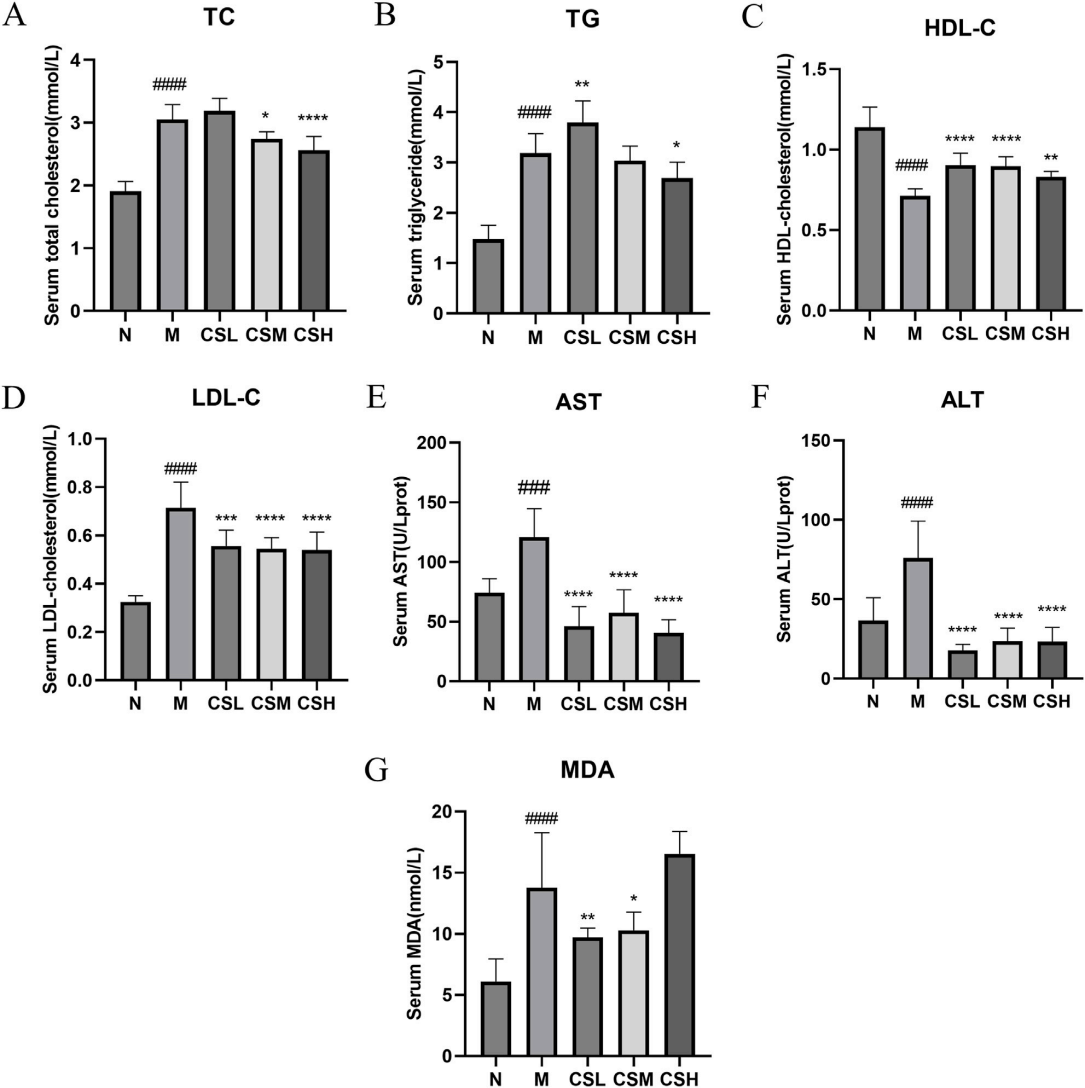
Effects of CSE on serum biochemical parameters in hyperlipidemic rats. **(A)** TC, **(B)** TG, **(C)** HDL-C, **(D)** LDL-C, **(E)** AST, **(F)** ALT, **(G)** MDA. Values were presented as mean ± SD (n = 8). vs. Group N: ###p < 0.001, ####p < 0.0001; vs. Group M: *p < 0.05, **p < 0.01, ***p < 0.001, ****p < 0.0001.

Compared to rats in the N group, rats in the M group exhibited significantly elevated levels of TC, TG, LDL-C, AST, ALT, and MDA (p < 0.001, p < 0.0001), along with a significant decrease in HDL-C levels (p < 0.0001), confirming successful establishment of a hyperlipidemia model. Following intervention with CSE, there was a significant improvement in the levels of TC, TG, HDL-C, LDL-C, AST, ALT, and MDA (p < 0.05, p < 0.01, p < 0.001, p < 0.0001) (Figures 5A-G), These results indicate that CSE effectively mitigates HFD-induced hyperlipidemia.

### 3.4 Effects of CSE on hepatic lipid accumulation in hyperlipidemic rats

**FIGURE 6:**
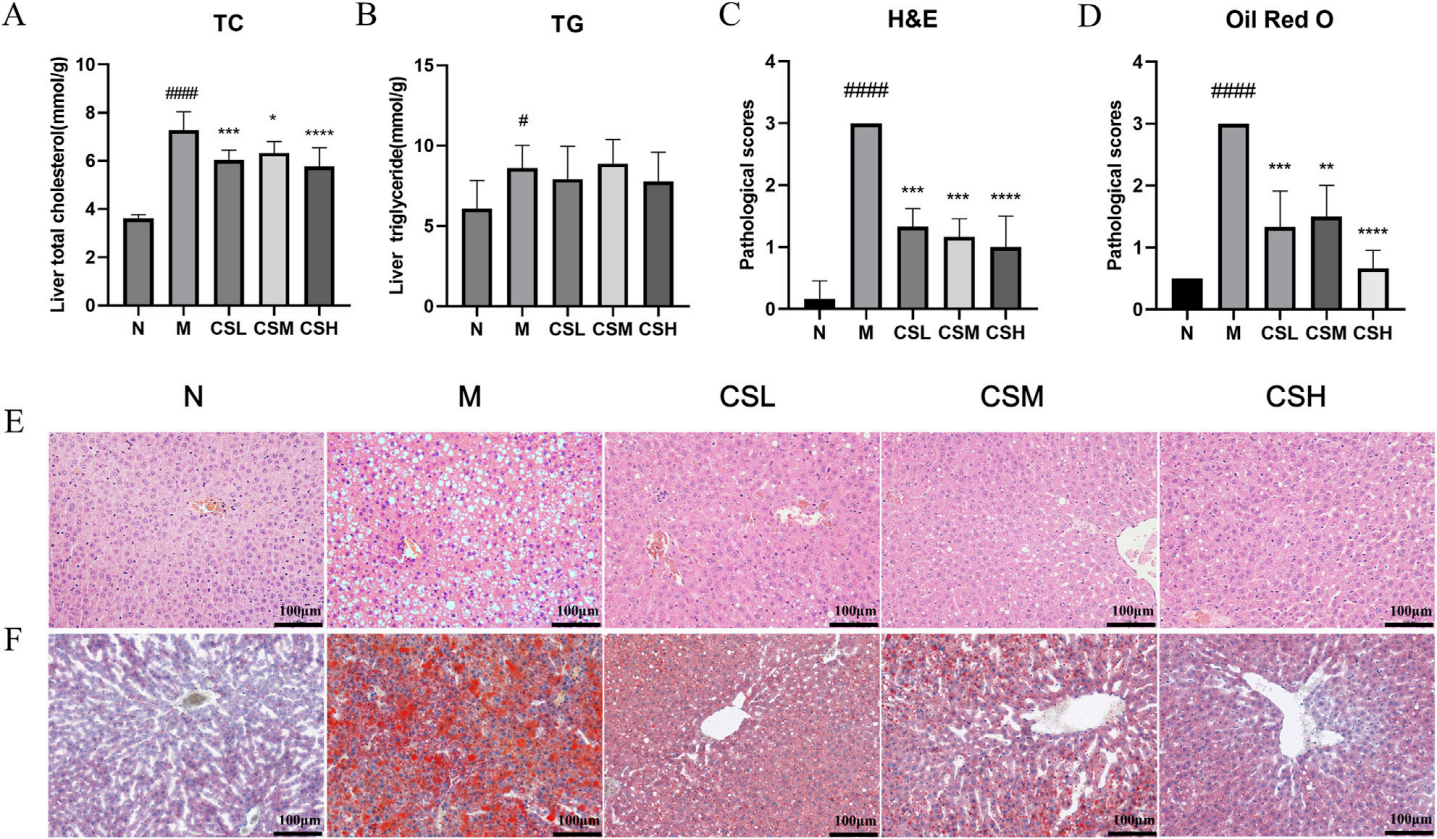
Effects of CSE on hepatic lipid lesions in hyperlipidemic rats. **(A)** Liver TC, **(B)** Liver TG, **(C)** Pathological scoring of H&E staining, **(D)** Pathological scoring of Oil Red O staining, **(E)** H&E staining, **(F)** Oil Red O staining. Values were presented as mean ± SD (n = 8). vs. Group N: ###p < 0.001, ####p < 0.0001; vs. Group M: *p < 0.05, **p < 0.01, ***p < 0.001, ****p < 0.0001.

We evaluated the impact of CSE on hepatic TC and TG levels to assess its efficacy in treating hyperlipidemia (Figures 6A, B). The TC and TG levels in rats of the M group were significantly higher than those in the N group (p < 0.05, p < 0.0001). Compared to the M group, treatment with CSE significantly reduced hepatic TC levels (p < 0.05, p < 0.001, p < 0.0001), whereas TG levels did not show significant changes.

H&E staining results (Figure 6E) revealed that in the N group, hepatocytes were well-organized with visible nuclei, intact hepatic lobular structure and no inflammatory cell infiltration. In contrast, rats in the M group exhibited enlarged and swollen hepatocytes, severe damage, ballooning of hepatocytes with displaced nuclei, and extensive hepatic steatosis. Following CSE intervention, there was significant improvement in hepatocyte morphology, evident by restored cell contour structures and reduced hepatic damage severity (Figure 6C), indicating that CSE has a certain therapeutic effect on hepatic steatosis.

Oil Red O staining results (Figure 6F) showed minimal lipid content in hepatic cells of the N group, considered as baseline. Conversely, the hepatic tissue from the M group rats were filled with abundant red lipid droplets, swollen hepatocytes, extensive lipid degeneration, and partial hepatocyte necrosis. Compared to the M group, treatment with CSE significantly reduced hepatic lipid accumulation, with decreased lipid droplets, improved hepatocyte structure, and the most pronounced effect observed in the high-dose group, approaching normal levels (Figure 6D). These results suggested that CSE can alleviate liver lipid aggregation and liver injury in hyperlipidemic rats.

### 3.5 Effects of CSE on gut microbiota in hyperlipidemic rats

**FIGURE 7:**
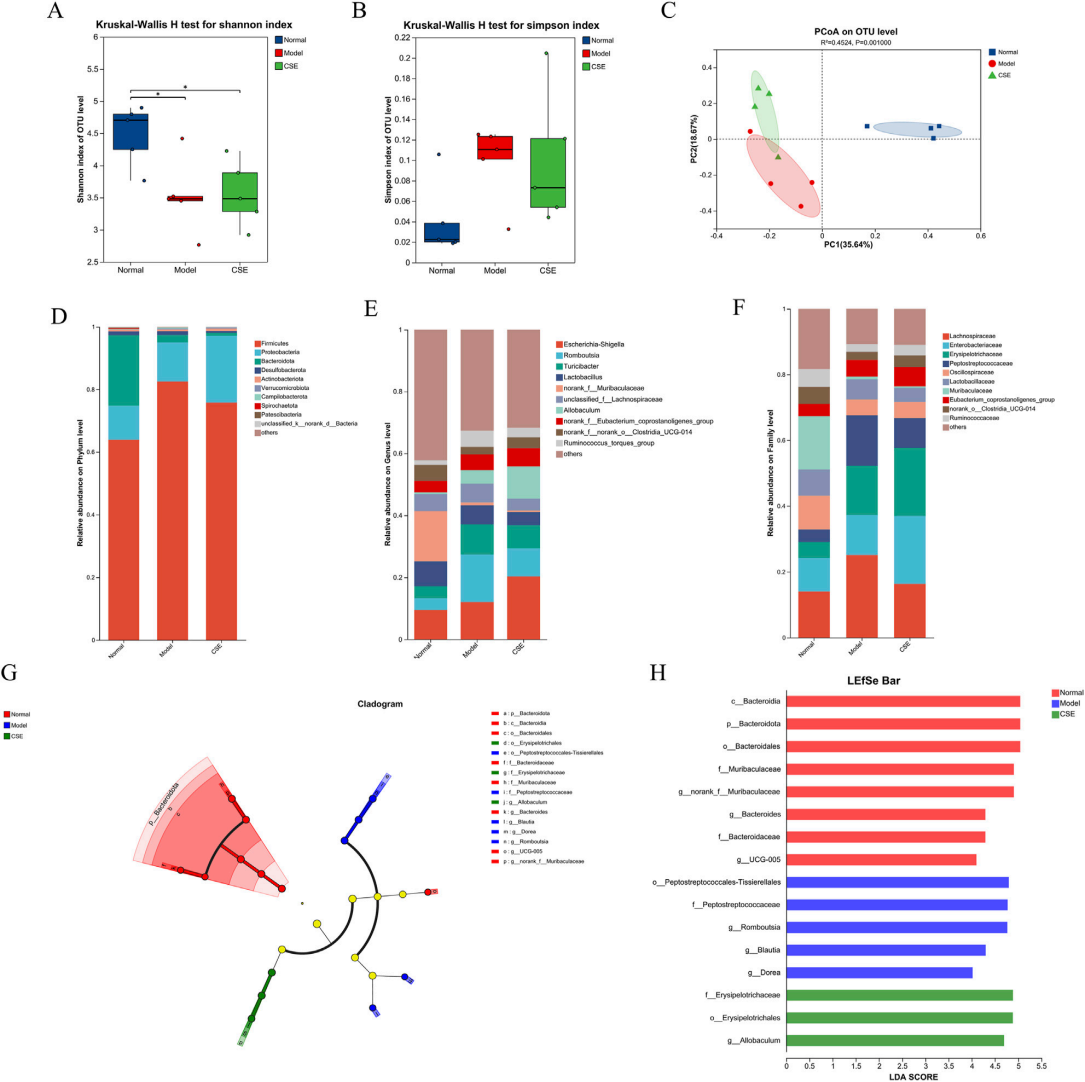
Effects of CSE on the gut microbiota in hyperlipidemic rats. **(A)** Shannon diversity index, **(B)** Simpson diversity index, **(C)** PCoA analysis, **(D)** Phylum-level classification abundance chart, **(E)** Genus-level classification abundance chart, **(F)** Family-level classification abundance chart, **(G)** LEfSe multi-level species hierarchical tree diagram, **(H)** LDA discrimination result table.

To evaluate the impact of CSE on the gut microbiota of hyperlipidemic rats, we conducted gene sequencing on fecal samples. Firstly, species diversity was assessed using the Shannon and Simpson indices (Xu et al., 2023) (Figures 7A,B). Compared to the N group, the M group exhibited a decrease in the Shannon index and an increase in the Simpson index, indicating microbiota dysbiosis induced by the HFD. Treatment with CSE partially mitigated these changes, increasing the diversity of gut microbiota. In addition, we analyzed the trend of gut microbiota in rat fecal samples using Principal co-ordinates analysis (PCoA) (Figure 7C). The results showed that the M and N groups were far apart from each other, indicating significant microbial differences between these two groups, while the CSE group was able to separate from the M group in the PC2 dimension, suggesting that CSE was able to regulate gut microbiota of rats.

We next examined the changes in microbiota composition at the phylum, family, and genus levels (Figures 7D–F). At the phylum level, *Firmicutes*, *Proteobacteria*, and *Bacteroidota* were the predominant phyla, collectively accounting for over 95% of the relative abundance. In the M group, *Firmicutes* abundance increased by 18.64%, becoming the dominant microbial population compared to the N group. However, CSE treatment reduced *Firmicutes* abundance by 6.77%, suggesting a potential corrective effect on the microbiota imbalance induced by the HFD.

At the family level HFD intervention led to an increase in the relative abundance of harmful bacteria such as Erysipelotrichaceae in the M group, while beneficial families like Oscillospiraceae and *Muribaculaceae* were reduced. CSE treatment did not produce significant changes in these bacterial populations, likely due to the complexity of microbiota interactions. Although CSE may have a broader impact on gut microbiota balance, it may not directly influence all microbial families.

Genus-level analysis revealed that *Romboutsia*, *Turicibacter*, and *Ruminococcus* were elevated in the M group, while *norank-f-Muribaculaceae* and *norank-f-norank-o-Clostridia-UCG-014* were significantly reduced. These findings indicate that HFD-induced dysbiosis was present in the gut microbiota of rats. CSE treatment mitigated these changes, suggesting a potential restoration of gut microbiota balance.

Further analysis using LEfSe (Linear Discriminant Analysis Effect Size) highlighted significant species differences (Figures 7G,H), with *Bacteroidota* enriched in the N group and differential impacts on *Peptostreptococcales* and Erysipelotrichaceae in the M and CSE groups. These results suggest that CSE administration alters the gut microbiota composition in hyperlipidemic rats, which may contribute to its anti-hyperlipidemia effects by modulating microbiota structure and diversity.

### 3.6 Metabolomic analysis results

#### 3.6.1 Methodological validation results

**FIGURE 8:**
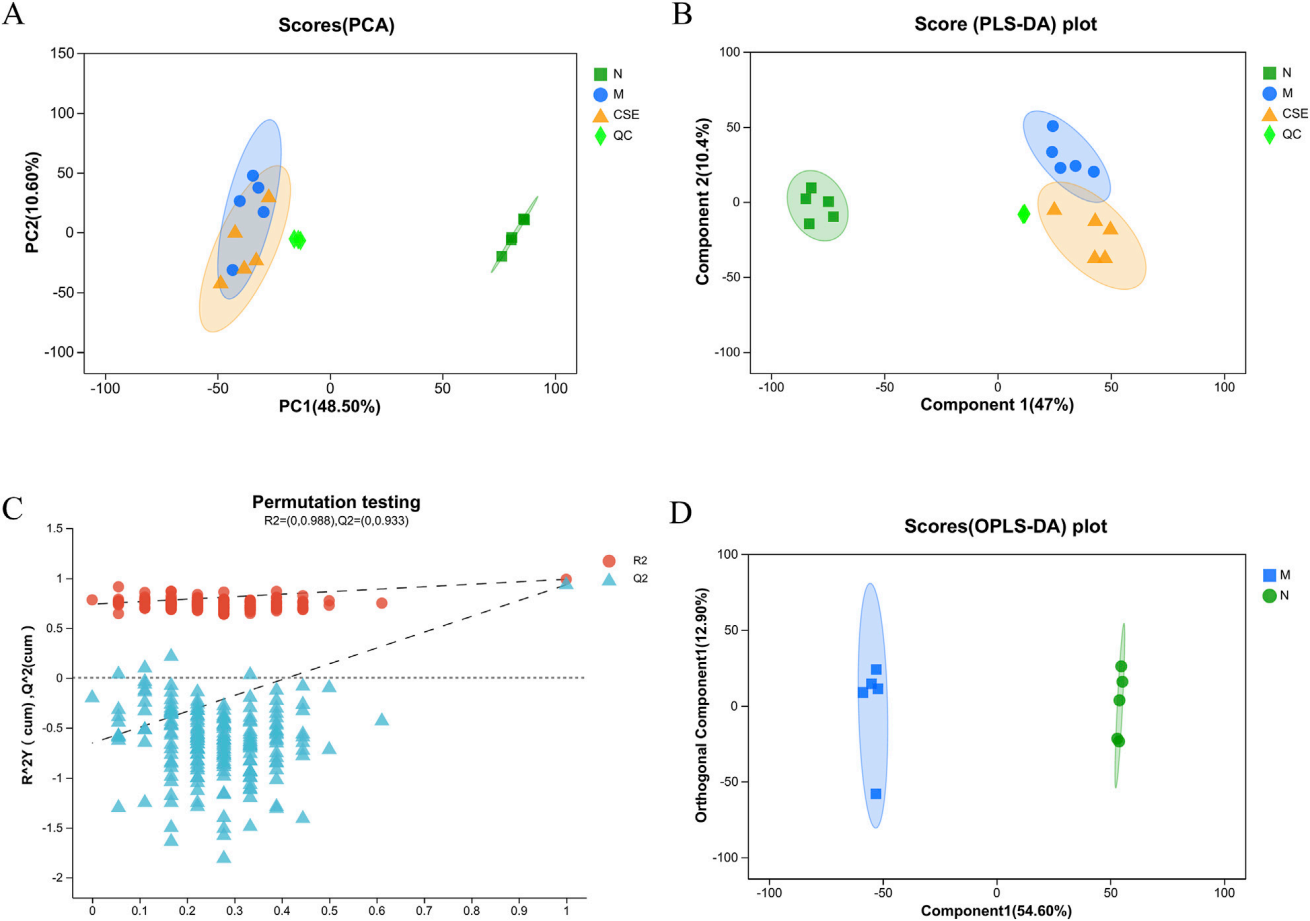
Pattern recognition plots of samples from the N group, M group, and CSE group rats. **(A)** PCA Score Plot **(B)** PLS-DA Score Plot, **(C)** PLS-DA Score Model Validation Plot, **(D)** OPLS-DA Score.

Principal Component Analysis (PCA) score plot (Figure 8A) demonstrated distinct clustering of samples from each group, indicating significant metabolic disruption in rats induced by HFD in the M group compared to the N group. Samples from the CSE group showed a trend towards the N group, suggesting a potential normalization of metabolic profiles with CSE treatment, although the inter-group differences were not statistically significant. Partial Least Squares Discriminant Analysis (PLS-DA) further confirmed substantial differences in endogenous metabolites among the groups (Figure 8B). The quality control was good, with stable system repeatability, ensuring reliable data analysis. The validation plot of the PLS-DA score model (Figure 8C) indicated adequate model reliability, as evidenced by lower R2 and Q2 values on the left side, and the Q2 regression line intersecting below the origin, indicating no overfitting. Orthogonal Partial Least Squares Discriminant Analysis (OPLS-DA) (Figure 8D) highlighted significant separation between the N and M groups, indicating profound metabolic dysregulation in hyperlipidemic rats. All samples fell within the 95% confidence interval, reinforcing the robustness of the metabolic differences observed.

#### 3.6.2 Differential metabolite and KEGG pathway enrichment analysis

**FIGURE 9:**
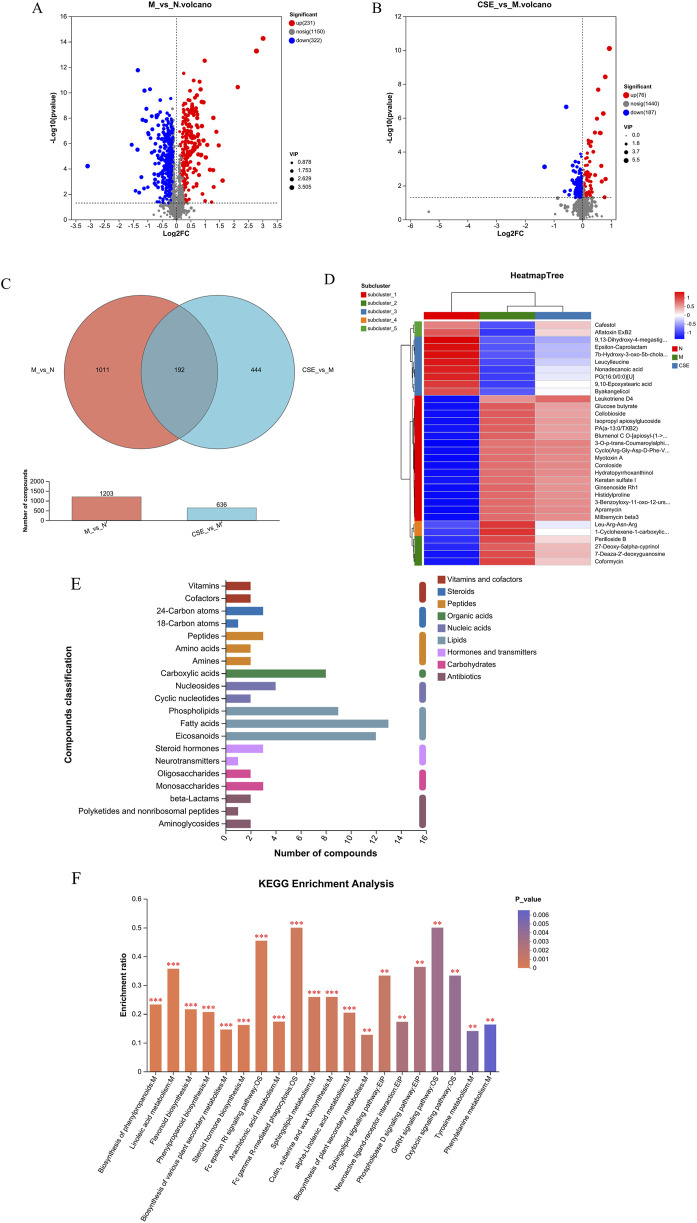
Analysis of differential metabolites and KEGG enrichment pathways. **(A)** Volcano plot of differences between M group and N group, **(B)** Volcano plot of differences between M group and CSE group, **(C)** Venn diagram, **(D)** Metabolite clustering analysis, **(E)** KEGG metabolites classification, **(F)** KEGG enrichment pathways.

Differential metabolite analysis was conducted comparing the N group and M group rats (VIP>1 combined with univariate analysis p < 0.05), revealing a total of 553 differential metabolites. Compared to the N group, the M group showed an increase in 231 metabolites and a decrease in 322 metabolites. Differential metabolite analysis was also performed on the M group and the group treated with CSE (VIP>1 combined with univariate analysis p < 0.05), identifying 263 differential metabolites, with 76 metabolites increased and 187 decreased in the CSE group relative to the M group (Figures 9A,B). A Venn diagram (Figure 9C) illustrated 192 common differential metabolites among the N group, M group, and CSE group. Cluster analysis of these metabolites indicated that the metabolic perturbations observed in the M group compared to the N group could be partially normalized by CSE administration (Figure 9D). According to the KEGG metabolites classification analysis (Figure 9E), lipids were the predominant class affected, particularly, fatty acid metabolites account for the largest. Further KEGG pathway enrichment analysis (Figure 9F) highlighted significant enrichment in the Fc gamma receptor-mediated phagocytosis pathway, indicating a potential mechanism through which CSE may exert its lipid-lowering effects.

### 3.7 Effects of CSE on expression levels of lipid metabolism and inflammation-related proteins

**FIGURE 10:**
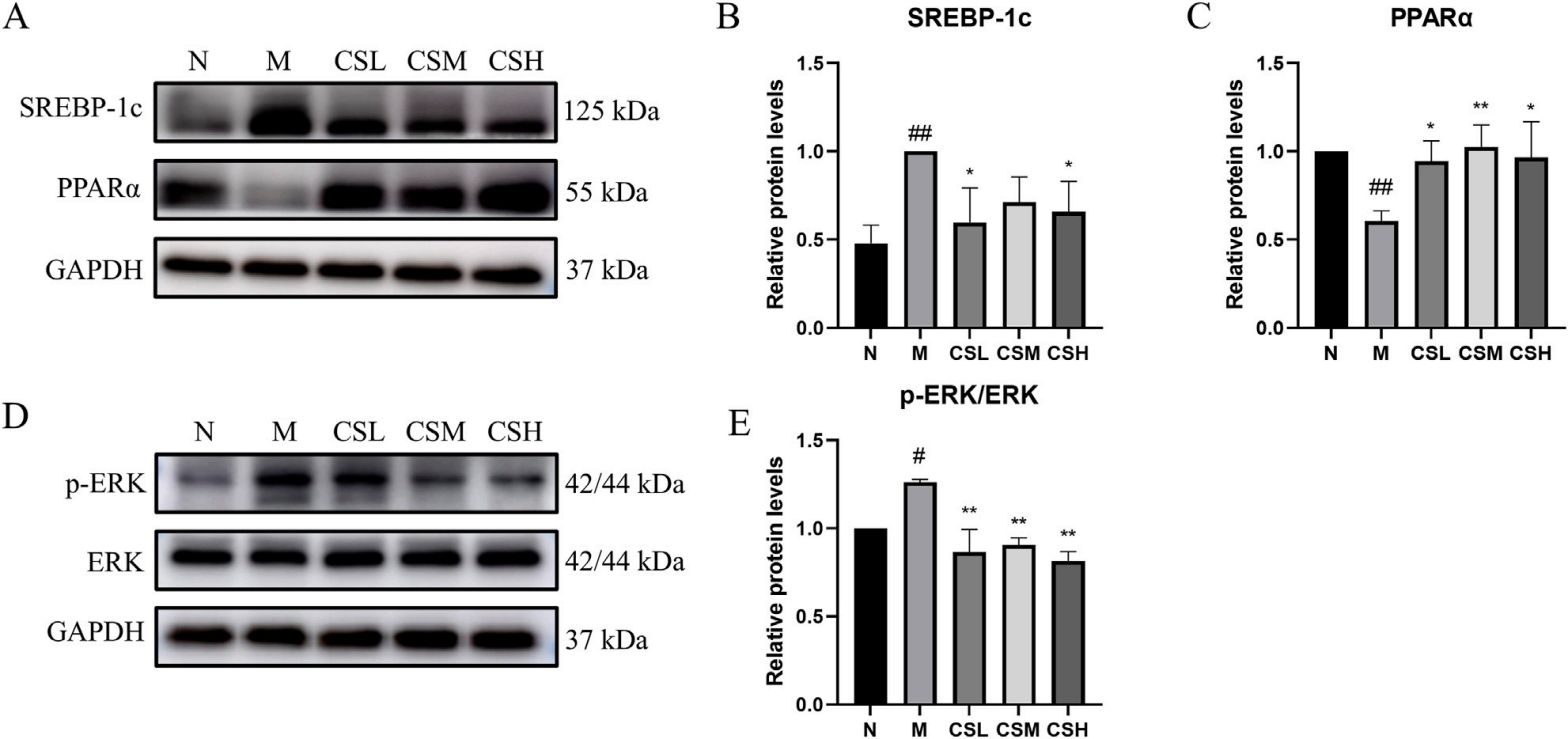
Effects of CSE on expression levels of lipid metabolism and inflammation-related proteins. **(A)** Expression levels of SREBP-1c protein and PPARα protein, **(B)** Relative protein levels of SREBP-1c, **(C)** Relative protein levels of PPARα, **(D)** Expression levels of p-ERK protein and ERK protein, **(E)** Relative protein levels of p-ERK/ERK. vs. Group N: #p < 0.05, ##p < 0.01; vs. Group M: *p < 0.05, **p < 0.01.

To further explore the mechanisms underlying the effects of CSE in hyperlipidemic rats, we evaluated the expression of proteins involved in lipid metabolism. Compared to the N group, the M group showed significantly increased SREBP-1c expression (p < 0.01) and reduced PPARα expression (p < 0.01). In contrast, CSE treatment significantly decreased SREBP-1c expression (p < 0.05) and increased PPARα expression (p < 0.05, p < 0.01) compared to the M group (Figures 10A-C). Additionally, we assessed the expression of ERK and its phosphorylated form. The M group exhibited significantly higher p-ERK levels than the N group (p < 0.05), while the CSE groups showed a significant reduction in p-ERK expression compared to the M group (p < 0.01) (Figures 10D, E). These findings suggested that CSE improved lipid metabolism and inflammation at the protein level.

## 4 Discussion

Hyperlipidemia is a prevalent global health issue and a risk factor for cardiovascular diseases, a leading cause of mortality (Husain et al., 2022). Traditional Chinese medicine (TCM) is increasingly recognized for its role in the treatment of hyperlipidemia. CS has gained recognition for its therapeutic potential in managing hyperlipidemia due to its widespread availability and documented safety and efficacy.

In this study, we firstly detected the main active metabolites in CSE by TLC, HPLC and HPTLC. In animal experiments, rats fed a HFD exhibited significant increases in body weight and liver indices compared to the N group, consistent with previous research findings (Hu et al., 2021). Intervention with CSE improved lipid levels, characterized by reduced TC, TG, and LDL-C levels, along with elevated HDL-C levels. Additionally, CSE administration lowered levels of AST and ALT, indicative of improved liver function, supported by histopathological evidence showing reduced hepatic lipid accumulation and diminished pathological damage, aligning with previous reports (Wang et al., 2019). Distinguishing itself from previous studies, our research focused on the impact of CSE on the gut microbiota of hyperlipidemic rats. Our results suggest that CSE enhances gut microbiota composition and diversity, potentially contributing to its lipid-lowering effects via mechanisms involving the Fc gamma receptor-mediated phagocytosis pathway.

The gut microbiota, often referred to as a “hidden organ,” plays a crucial role in human health. At the phylum level, the increase in *Firmicutes* and decrease in *Bacteroidetes* are important indicators of hyperlipidemia (Lu et al., 2022). *Firmicutes* species, known for their ability to produce butyrate, are associated with increased fat and energy absorption in obese individuals (Gomes et al., 2018). Rats fed a HFD exhibited an increase in *Firmicutes* levels, leading to gut microbiota dysbiosis. Therefore, the anti-hyperlipidemic effect of CSE may be achieved by modulating *Firmicutes* abundance. At the family level, compared to the N group, the M group exhibited elevated levels of Lachnospiraceae, Erysipelotrichaceae, Peptostreptococcaceae, and *Eubacterium*. While *Eubacterium* is generally considered beneficial, its increased levels have been associated with obesity in various studies (Verdam et al., 2013), consistent with the observed weight gain in HFD-fed rats in our study. However, after CSE intervention, we did not observe a significant reversal of these changes at the family level. This may be due to the fact that HFD-induced dysbiosis in the hyperlipidemic rat model might not be fully reversed by the drug intervention, reflecting the complex interactions between the drug and gut microbiota. Nonetheless, when considered alongside other indicators, these findings suggest that CSE exerts systemic benefits beyond alterations in gut microbiota alone.

Untargeted metabolomics offers a powerful tool for understanding the mechanisms underlying the therapeutic effect of CSE in treating hyperlipidemia induced by HFD. We identified a total of 192 differential metabolites by metabolomics analysis. And through further analysis of these differential metabolites, we classified these metabolites according to their metabolic pathways. This classification provides insights into the major classes of metabolites influenced by CSE treatment and their potential roles in modulating hyperlipidemia. We found that lipids constituted the largest proportion of the identified metabolites. These lipids were further categorized into Phospholipids, Fatty acids and Eicosanoids. This distribution highlighted the central role of lipid metabolism in the response to CSE treatment. Further analysis using KEGG pathway enrichment revealed Fc gamma receptor-mediated phagocytosis as significantly enriched upon CSE treatment. Fcγ receptors (FcγRs) play a crucial role in the immune system by recognizing and binding to the Fc region of immunoglobulin G (IgG). These receptors were classified into different types, such as FcγRI, FcγRII, and FcγRIII, each with distinct functions in various immune cells. By binding to antibodies, FcγRs modulate immune cell activity and influence immune responses (Osorio et al., 2023). They are key players in antibody-dependent immune mechanisms, including enhancing phagocytosis, mediating antibody-dependent cytotoxicity (ADCC), and activating the complement system (Musolino et al., 2022). FcγRs can promote the formation of foam cells by mediating macrophage phagocytosis of oxidized low-density lipoproteins (ox-LDL), which is central to the promotion of the development and progression of atherosclerosis (Ng et al., 2015; Hernández-Vargas et al., 2006).

The liver, a major organ involved in lipid metabolism, is influenced by immune system activity through various pathways. In this study, KEGG metabolites classification revealed that the differentially expressed metabolites were predominantly lipids, with fatty acids representing the largest proportion. Consequently, we assessed the expression of two key proteins involved in fatty acid metabolism in the liver: SREBP-1c and PPARα.

SREBPs are a family of transcription factors that regulate lipid homeostasis by modulating a series of enzymes required for the synthesis of endogenous cholesterol, fatty acids, and phospholipids. Among them, SREBP-1c is the primary regulator of hepatic fatty acid and triglyceride synthesis. A HFD increases SREBP-1c expression, promoting fatty acid and triglyceride synthesis, which elevates plasma triglyceride and cholesterol levels and leads to hyperlipidemia. Our results showed that CSE treatment inhibited lipid synthesis by down-regulating SREBP-1c expression.

PPARα, a key regulator of lipid metabolism, is highly expressed in the liver and activates the expression of enzymes involved in fatty acid β-oxidation and bile acid metabolism. We observed that PPARα expression was significantly reduced in the liver of hyperlipidemic rats, indicating that an HFD impairs fatty acid β-oxidation and bile acid metabolism, leading to dysregulated triglyceride and cholesterol metabolism. Treatment with CSE significantly upregulated PPARα expression, promoting fatty acid oxidation and bile acid metabolism, thereby improving triglyceride and cholesterol homeostasis in the liver. In certain metabolic diseases, abnormal lipid metabolism can also lead to dysregulation of the immune system, thereby exacerbating inflammatory responses. For example, in obesity, a large number of immune cells appear in the adipose tissue, and the abundance of immune cells increases dramatically with the increase in adipose tissue (de Heredia et al., 2012; Kawai et al., 2021). Macrophages, which shift from an anti-inflammatory M2 type to a pro-inflammatory M1 type, not only drive the immune response but also disrupt lipid metabolism, contributing to insulin resistance, fat accumulation, and a more intense inflammatory response.

Peiseler et al. (2022) demonstrated that hepatic fat overload induces lipotoxicity, triggering oxidative stress, ROS production, and mitochondrial damage. This results in the release of pro-inflammatory mediators from stressed hepatocytes, which activate and recruit immune cells, exacerbating liver damage, promoting cell death, and inducing senescence, thereby amplifying the immune response. In response to tissue injury, pattern recognition receptors (PRRs) in innate immune cells are activated, initiating the MAPK signaling pathway (Arthur and Ley, 2013). MAPK activation subsequently upregulates genes that regulate the inflammatory response. In our study, we observed a significant increase in p-ERK in the livers of hyperlipidemic rats, indicating hepatic inflammation, likely due to excessive activation of the MAPK signaling pathway (Wu et al., 2024). CSE effectively inhibits p-ERK expression and reduces hepatic inflammation.

In summary, this study explores the therapeutic mechanisms of CSE in alleviating hyperlipidemia induced by a high-fat diet in rats. Our findings demonstrate that CSE effectively reduces serum lipid levels (TC, TG, LDL-C) and increases HDL-C, while improving liver function and alleviating hepatic steatosis. Additionally, CSE modulates the gut microbiota, notably decreasing *Firmicutes* abundance, which is linked to obesity and lipid dysregulation. Metabolomics analysis reveals significant alterations in lipid metabolism, particularly through the FcγR-mediated phagocytosis pathway, which is involved in lipid-related inflammation. CSE downregulates hepatic SREBP-1c (inhibiting lipid synthesis) and upregulates PPARα (promoting fatty acid β-oxidation), restoring lipid homeostasis. Moreover, CSE reduces hepatic inflammation by inhibiting MAPK/ERK signaling, which is associated with FcγR-mediated immune activation. These results suggest that CSE, by regulating lipid metabolism and immune pathways, offers a promising natural treatment for hyperlipidemia and its inflammatory complications, providing a novel approach to metabolic disorders in Traditional Chinese Medicine.

## Data Availability

The data presented in the study are deposited in the NCBI repository, accession number PRJNA1251840.
